# Measuring, Modeling, and Forecasting the Mental Wealth of Nations

**DOI:** 10.3389/fpubh.2022.879183

**Published:** 2022-07-28

**Authors:** Jo-An Occhipinti, John Buchanan, Adam Skinner, Yun Ju C. Song, Kristen Tran, Sebastian Rosenberg, Allan Fels, P. Murali Doraiswamy, Petra Meier, Ante Prodan, Ian B. Hickie

**Affiliations:** ^1^Faculty of Medicine and Health, Brain and Mind Centre, University of Sydney, Sydney, NSW, Australia; ^2^Computer Simulation and Advanced Research Technologies, Sydney, NSW, Australia; ^3^Mental Wealth Initiative, University of Sydney, Sydney, NSW, Australia; ^4^Melbourne Institute of Applied Economic and Social Research, Melbourne Law School, University of Melbourne, Melbourne, VIC, Australia; ^5^Departments of Psychiatry and Medicine, Duke University School of Medicine, Duke University, Durham, NC, United States; ^6^Systems Science in Public Health, University of Glasgow, Glasgow, United Kingdom; ^7^School of Computer, Data and Mathematical Sciences, Western Sydney University, Penrith, NSW, Australia

**Keywords:** mental health, wellbeing, gross domestic product (GDP), mental capital, policy analysis, systems modeling, simulation

## Abstract

The COVID-19 pandemic has exposed the deep links and fragility of economic, health and social systems. Discussions of reconstruction include renewed interest in moving beyond GDP and recognizing “human capital”, “brain capital”, “mental capital”, and “wellbeing” as assets fundamental to economic reimagining, productivity, and prosperity. This paper describes how the conceptualization of Mental Wealth provides an important framing for measuring and shaping social and economic renewal to underpin healthy, productive, resilient, and thriving communities. We propose a transdisciplinary application of systems modeling to forecast a nation's Mental Wealth and understand the extent to which policy-mediated changes in economic, social, and health sectors could enhance collective mental health and wellbeing, social cohesion, and national prosperity. Specifically, simulation will allow comparison of the projected impacts of a range of cross-sector strategies (education sector, mental health system, labor market, and macroeconomic reforms) on GDP and national Mental Wealth, and provide decision support capability for future investments and actions to foster Mental Wealth. Finally, this paper introduces the Mental Wealth Initiative that is harnessing complex systems science to examine the interrelationships between social, commercial, and structural determinants of mental health and wellbeing, and working to empirically challenge the notion that fostering universal social prosperity is at odds with economic and commercial interests.

## Introduction

The COVID-19 pandemic has exposed the fragility of economic, health and social systems. Globally, at the time of writing, the pandemic has claimed over 5.6 million lives (1), overwhelmed health systems (2), precipitated the worst global recession in nearly a century (3), and pushed 97 million more people into poverty (4). Importantly, the pandemic has also brought into sharper ocus the consequence of unequal and divided societies. The physical, social, and economic impacts of COVID-19 have disproportionately affected the most vulnerable (5, 6). Within nations, factors such as poverty, poor housing conditions and overcrowding, and inadequate community infrastructure, hinder the ability to respond individually and collectively to crises (7–9). Poverty is also associated with increased rates of depression, anxiety, and other common mental illnesses which further exacerbate vulnerability (10). Such social vulnerability has been associated with greater COVID-19 incidence and mortality (11). Between nations, unequal vaccine distribution has hampered global efforts to suppress the emergence of new variants and bring an end to the pandemic. In addition, over recent decades, the economic and social development of nations has commonly prioritized the individual over the collective, exacerbating inequalities and eroding social cohesion (12), which has further undermined public health responses to the pandemic. Nations giving primacy to more individualistic (vs. collective) policies have generally endured higher COVID-19 cases, mortality, and public non-adherence to prevention measures (13–15). Tensions between individual rights and civil responsibility have divided communities and exacerbated anti-government sentiment, resulting in increased civil unrest and political instability (16). The profound structural weaknesses exposed by the pandemic and the lessons it has taught us on the deep links between health and economic development, and the socio-political determinants shaping inequalities in both, have left many questioning the merits of “returning to normal.” Crises commonly intensify pre-existing, often regressive, trajectories of development (17–20). The pandemic provided no exception, catalyzing the redistribution of wealth to the wealthy and further exacerbating disadvantage (21, 22). This has fueled renewed discussion about the need for reconstruction of economic, social, and health systems in ways that will deliver healthier, more equal and resilient societies capable of collectively responding to looming global challenges (23).

While crises represent a pivotal moment for change, progress is far from guaranteed. The paradigm shift required to support social and economic reimagining will require a wider economic lens than is currently prevalent, as well as new metrics against which to assess progress and national prosperity. Mazzucato (24), in her exploration of centuries of economic evolution, points out that changing ideas of what constitutes “value” in the economy has been instrumental in shifting the “production boundary” over time, re-defining what is measured, and ultimately shaping change in economic and social development. Post-pandemic reconstruction will therefore require a fundamental rethink about what we value, and how we conceptualize, measure, model, and forecast national prosperity. This will be vital to inform priorities concerned with more inclusive and sustainable economic and social development that fosters community and system resilience. This paper outlines the conceptualization and measurement of Mental Wealth, a wider lens against which we can assess social and economic progress. Additionally, we propose the implementation of complex systems modeling to forecast the Mental Wealth of nations and understand the extent to which policy-mediated changes in the economic and social environment could enhance the trajectory of national prosperity and build resilience against future threats.

### I. Problems and Advances in Generating and Estimating National Prosperity

Recent decades have seen increasing recognition of the limitations and perversions of the prevailing orthodoxies of “small government”, greater promotion of market forms of economic and social organization, and accompanying cultural shift toward individualism (25). A legacy of this orthodoxy has been a preoccupation with maximizing the production (and consumption) of goods and services at the lowest price. This provides the basis for defining national prosperity and higher material standards of living for all (26). In reality, however, this fosters perverse incentives in the pursuit of lower costs of production and increases in labor productivity. Paradoxically, this pursuit, in the interests of growing the economy to increase national prosperity, undermines collective wellbeing by creating a tradeoff between consumer and worker wellbeing. It devalues and actively excludes those not contributing to “the formal economy” (e.g., caregivers, volunteers, or those who are unemployed, old, or disabled), deepens inequality, and erodes mental health in advanced economies (25–28). Through the current economic lens, population wellbeing is of interest only to the extent that it preserves labor productivity, giving rise to the deficit-based approach where compelling arguments for addressing population health and wellbeing are made on the basis of estimating the cost of lost productivity.

Efforts are being made to address imbalances which have emerged from the 30-year experiment with small government and increased marketization. Some of these efforts primarily retain the existing economic frame (production boundary) (29, 30), while others seek to broaden notions of “the economy” and what constitutes a prosperous society (26, 31–34) (Box 1). Consistent across them is recognition that a fundamental shift in our societal trajectory is essential to achieving healthier individuals, functional families, more cohesive, resilient communities and societies, and economies more capable of meeting new national and global challenges. These positive outcomes would ultimately reduce the impost on governments.

Box 1 **Exemplars in reconceptualizing “the economy”.**

□ The **Inclusive economy** (29, 30, 35)—working to address income inequality, uneven distribution of the tax burden between capital and labor, and financial mechanisms (such as tax havens) that contribute to inequality. It seeks to deliver on equity and inclusion in an economy's design; in the access of resources that enable participation in the economy (including health and education), and in distribution of the benefits generated in the economy (including income, assets, and goods and services).
□ The **Foundational economy** (31)—seeks to better recognize the importance of health, care, education, housing, utilities and food supply as drivers of welfare and the basis of citizenship. The European-based Foundational economy alliance recognizes the supply of these basic services for all citizens as having a greater contribution to wellbeing than individual consumption. This economic paradigm aims to widen the lens of policymakers in understanding the value of investing in the infrastructures of everyday life.
□ The **Civil economy** paradigm (26)—seeks to counter “anthropological reductionism” where profit maximization is socially harmful and instead create value from “civic fertility” and accumulation of a multi-dimensional stock of (spiritual, economic, relational, environmental, and cultural) goods enjoyed by a community.
□ The **Wellbeing economy** (34)—emphasizes importance of a harmonious relationship between society and nature, a fair distribution of resources, and healthy and resilient communities (including collective wellbeing). The wellbeing economy encompasses a diverse array of ideas and actions aimed at advancing social wellbeing through three basic principles. It recognizes that people need to restore a harmonious relationship between society and nature, enjoy a fair distribution of resources, and live in healthy and resilient communities.
□ The **Green economy** (32) and **Doughnut economics** (33)—frameworks for sustainable development recognize the importance of balancing social foundations and ecological boundaries associated with economic development and national prosperity. These paradigms promote a fundamental transition toward more sustainable modes of production and consumption.

In parallel with these endeavors to rebalance economic priorities have been initiatives directed at expanding the way in which we estimate national prosperity. The key concern of these literatures is overcoming the problem of GDP's status as the predominant measure of economic success. GDP is, historically speaking, a relatively new creation. Following the deep crises of the Great Depression and World War II new ways of estimating national prosperity emerged (36). Beginning in 1920s Germany and emerging independently in the US and UK in the 1930s and 1940s, systems of national accounts were devised to not just measure the economy—but help reconstruct it in a different image (37). The form of national accounting that ultimately prevailed was based on the legacies of planning for the UK and US war economies. These were underpinned by Keynes' deep insight that the modern economy is best understood as not primarily involving the exchange of real goods and services—but rather as a complex entity shaped profoundly by the dynamics of increasing productive capacity and money. In such a system there is no mechanism that automatically ensures a balance between what the economy is capable of producing (supply) and demand (38). At their core, systems of national accounts embody this reality: that we live in a money production economy. The real power of GDP, the key measure of performance arising from the national accounts, is that it embodies these deep insights. At its most basic, when expressed in terms of demand, GDP can be summarized in the basic accounting identity:


(1) $$\begin{array}{l} \text{ GDP }=\text{ C }+\text{ I }+\text{ G }+\text{NX} \end{array}$$

where *C* is consumption or private consumer spending, *I* is the sum of a country's investments on capital equipment, *G* is total government expenditures, inventories, and housing, and NX is net exports (39). Managing the economy from the late 1940s to the early 1970s on the basis of these aggregates, policy makers in the advanced capitalist economies delivered the greatest improvements in material living standards in history. Even as Keynesian inspired policies lost ascendancy after that period, the enduring legacy of these concepts survived. They provided the estimates that guided the massive fiscal and monetary responses to the deep crises of 2008/09 and 2020/21 (40–42).

While the systems of national accounts represent an exceptional human achievement, GDP is far from an adequate indicator of national prosperity. It was never designed as a national wellbeing measure but is often misused as such. Ironically, the source of GDP's strength is also the basis of its weakness: preoccupation with monetized production. Officially “GDP is a measure of the value of the final goods and services produced within a country at a given time period” [(43), p. 16]. Mazzucato [(24), p. 100] is more direct in pointing out that within the current production boundary “any activity that can be exchanged for a price counts as adding to GDP”. A host of problems arise from this foundation. The two most powerful criticisms arise from concerns about distribution and what does (and does not) get recognized as “value adding” activity. Distributional issues are important because it is increasingly recognized GDP growth alone means little if the gains are not widely shared (44, 45). The problems arising from what goods and activities are included and excluded within GDP are arguably even more profound. Of starkest concern is the exclusion of activities beyond the market sphere. Government is primarily recognized as a source of “consumption” and its investment role neglected [(24), Ch 8]. A host of non-market, non-government activities are ignored altogether (37, 43, 46–48). Prime amongst these are care activities in the household and important social institutions providing vital community (micro) infrastructures that are essential for community cohesion and resilience (e.g., volunteer fire brigades, surf life-saving clubs, community centers, and sporting organizations). Even within the market sphere there are problems. Replacing infrastructure damaged by natural disasters can boost GDP but does not signify greater national prosperity. The output of a company causing pollution increases GDP and will then boost GDP even more if it is then obliged to pay another firm to clean it up. The OECD refers to this as the problem of “regrettables” [(43), p. 17].

Problems of this nature have been recognized since the inception of the national accounts (24, 36, 37). Since the late 1980s, and especially in the last two decades, researchers from a range of settings have embarked on concerted programs to get better measures of national wellbeing and prosperity. Supplementary Table S1 summarizes six major research programs. These contributions fall into three broad categories summarized as follows:

(a) **Adding data items that make up for what the national accounts leave out**. The *OECD's Measuring Wellbeing* initiative has been operating for over a decade (49). It has now amassed vast time series data on the quality of life, material conditions, and resources for future wellbeing of OECD member nations. The United Nations Development Program has been building its Human Development Index for over three decades (50, 51). Initially its focus was on life expectancy, education, and material living standards. More recently it added comprehensive information on different dimensions of inequality and how these impact on rankings of human development (52).
(b) **Reconfiguring data items to make better use of latent distributional information in the national accounts and associated data collections**. In 2014, Thomas Picketty released a landmark study on *Capital in the twentieth century*. This built on years of work he and colleagues had undertaken to document changes in the distribution of income, both functional (balance between capital and labor) and personal (spread across the population) (53). This has highlighted that understanding growth on its own provides at best a partial understanding of national prosperity. How it is distributed has changed dramatically over time within countries. Researchers analyzing the foundational economy have also reconfigured data collected around basic categories to generate novel insights (54). Their liveability index, for example, presents how residual household income after expeneses on material foundations of life are controlled for. This reveals that places with high GDP per capita (e.g., London) often have a lower material standard of living than those in places of lower GDP per capita (e.g., rural Wales) once the costs of housing, transport and utilities are considered.
(c) **Refining the core data items that currently comprise GDP**. The above two responses leave the core categories of the national accounts unchanged. They either add additional ones to provide supplementary indices or reconfigure them to generate new insights. Several research programs have been more thoroughgoing. The *Successful Societies* research program brought health and social researchers together to think through the meaning of “success” (55). Two findings were particularly powerful. First, they have identified the importance of distinguishing material from social conditions when examining wellbeing. Second, success requires consideration not just of resources and capacities held by individuals and groups—it also requires consideration of the constraints facing them. The Mental Wealth research program has generated similar findings. Its analysis commenced by noting the importance of nurturing mental as well as material wealth (56, 57).

These developments have been remarkably rich in generating powerful new knowledge. Compared to just two decades ago researchers now have a much better understanding of measures of living standards than that provided by the traditional metric of GDP. The impact of these research programs on policy priorities, however, has been subdued. Growth in GDP as conventionally understood remains the pre-eminent public policy priority. Factors beyond the research community have clearly been the prime cause for this situation. There are, however, limitations in the design of most of the alternative approaches to estimating national prosperity that have been proposed. Most have been devoted to getting “beyond GDP”. This orientation has limited the challenge to GDP as conventionally understood in two ways. First, the compilation of additional data has mainly concerned supply side issues, especially factors concerning labor supply (e.g., longevity, educational attainment). Second, it leaves the conventional way of measuring demand essentially untouched. Gross National Income (GNI) is explicitly embedded in the Human Development Index. The OECD's Wellbeing Framework explicitly lists GDP as a separate entity surrounded by the additional data items making up its indices. Such an approach has generated remarkably rich, complementary data sets—but some of the greatest limitations of GDP are problems of misspecification (i.e., of how what is included is defined), not just a problem of what has been left out.

If we are interested in arriving at better estimates of national prosperity it is important that the achievements of the recent programs to enrich estimates of GDP of the kind summarized in Supplementary Table S1 are continued. But we do not need simply more of the same. As noted earlier, the design principles of the national accounts contained kernels of profound insight about the character of living in a money production economy. We need to build on the content of the demand side data items concerning consumption and investment and renovate how they are defined. Both the *Successful Societies* and Mental Wealth literatures highlight the importance of thinking through core categories concerning the material and the social or “mental” dimensions of activity. The particular attraction of the Mental Wealth conceptualization, however, is that its framing of issues resonates deeply with the importance given to demand side factors that initially informed the construction of the national accounts. Simply boosting mental capital on its own is unhelpful if such capital deepening is not harnessed through use. This is why the Mental Wealth conceptualization provides such a powerful reference point for thinking through how to generate better estimates of national prosperity: it is concerned with better defining conditions of demand—as well as conditions of supply.

### II. The Origins of Mental Wealth

The importance of labor productivity to economic growth and development gave rise to Human Capital Theory in the 1960s, positing that investments in education and training can increase innovation, creativity, and ultimately the productive capacity of individuals (58). While the theory has long had its critics (59–61), the post-modern shift toward knowledge- and service-based economies is refocusing attention on human aptitude as a critical driver of productivity, competitiveness, and prosperity (57, 62). Concepts of “brain capital” (63) and “mental capital” (56) are being emphasized as fundamental assets for economic reimagining. It is argued that increased automation has given rise to a “Brain Economy” where “*most new jobs demand cognitive, emotional, and social, not manual, skills and where innovation is a tangible ‘deliverable' of employee productivity*” (63). This thinking has given rise to the concept of Mental Wealth first outlined by Beddington et al. in their 2008 paper in *Nature* (56). This paper summarized insights from the Foresight Mental Capital and Wellbeing Project, a 2-year project by the UK Government Office for Science working in collaboration with more than 450 experts and stakeholders across 16 countries, to synthesize existing evidence and knowledge of the factors that influence mental development and wellbeing across the lifecourse (57). This work highlights mental capital, mental health, and wellbeing as fundamental to the ability to achieve individual and collective potential, social cohesion, and national prosperity (57).

The Foresight Mental Capital and Wellbeing Project defined **Mental capital** as “*the totality of an individual's cognitive and emotional resources…”* which includes “*cognitive capability, flexibility and efficiency of learning, emotional intelligence (for example, empathy and social cognition), and resilience in the face of stress”* (64). Unlike fiscal capital “*it* [mental capital] *is not depleted by ‘spending' it*” (64); indeed deploying mental capital well is essential for its growth. **Mental health and wellbeing** mediates the ability to acquire and deploy mental capital and is defined as “*a dynamic state in which the individual is able to develop their potential, work productively and creatively, build strong and positive relationships with others, and contribute to their community”* (57). Therefore, mental health and wellbeing are not simply important in their own right but are a critical element for expanding and deploying mental capital for national and community development. The Foresight Mental Capital and Wellbeing Project provided rich consideration of how mental capital accumulated or deteriorated over the lifecourse and was expressed most strongly at the individual level (57). Less developed were considerations of the broader social settings shaping collective wellbeing and civil contribution, and the work was silent on methods to quantify the Mental Wealth of nations.

Those working in the field of mental health research and clinical practice have long known the centrality of mental health and wellbeing to social and vocational functioning (65–67), and conversely the mental health benefits afforded by gainful employment in modern societies where this is an expectation (68). Nonetheless, historically there has been an underappreciation of, and underinvestment in, mental health and wellbeing. Mental health is one of the most neglected areas of public health; even prior to the pandemic, mental disorders were among the leading causes of disability globally (69), while suicide is among the leading causes of years of life lost in high income countries (70, 71). Despite this burden, it is estimated that currently only two per cent of government health budgets globally are allocated to mental health (72). Despite growing attention and action in recent years by the World Health Organisation (73), the United Nations (74), the World Bank (75) and other leading global development organizations to improve mental health outcomes and enhance mental assets, progress thus far has been disappointing (76).

Nonetheless, investments are being made at an unprecedented scale in the wake of the pandemic. The World Bank has underscored the importance of governments investing in the human capital of their citizens and has committed up to US$160 billion to help countries reconstruct stronger, more equitable and resilient economic and social systems (75). Individual governments around the world are investing significant sums in stimulus packages. However, without operationalization of the concept of Mental Wealth to guide these investments there remains the risk of an overemphasis on more traditional physical infrastructure investment or accelerated capital spending for economic stimulus. Additionally, without a measure of Mental Wealth there is no basis for understanding:

- The extent to which investments and actions contributed to improving this broader measure of national prosperity
- The best conditions for fostering a nation's Mental Wealth—especially within the community and labor market
- It is unclear how best to allocate funding and resources across the drivers of Mental Wealth to deliver the greatest national benefit
- In operationalizing Mental Wealth there is uncertainty around how best to guard against the stark inequalities that have emerged as a result of current metrics, especially those built around GDP and traditional measures of human capital such as educational attainment. For example, too great a focus on mental capital while retaining the current economic frame (boundary) risks movement toward a socially polarizing meritocratic society (77–79) in the ongoing service of growth in labor productivity and GDP. It can place undue emphasis on social mobility (80) and undervalues those making unpaid contributions to society [hereafter termed “social productivity” (81)].

Addressing these challenges is the primary preoccupation of Australia's new Mental Wealth Initiative.

### III. The Mental Wealth Initiative

The Mental Wealth Initiative (MWI) is a multi-faculty enterprise of the University of Sydney's Brain and Mind Centre in partnership with the University of Sydney Business School, and in collaboration with research leaders and innovators across the Faculty of Medicine & Health, the School of Economics, and the Sydney Law School. The MWI is also working with external leaders across academia, government, business, mental health and social policy, and communities, to measure, model, and forecast the Mental Wealth of nations. By doing this, the MWI will identify and promote policy opportunities to foster Mental Wealth. The MWI enjoys the support of leading Australian economists and politicians, and international collaborators including the World Economic Forum's Global Future Council on Mental Health; the UK based SIPHER Consortium applying systems science in public health and economic research; and CSART, an international alliance of centers of excellence in systems modeling, simulation, and global health.

While the MWI has its roots amongst mental and population health experts, it embraces a range of disciplines within the formal, natural, and social sciences, economics, business, and the humanities. Their common concern is “how to achieve the best possible mental development and mental wellbeing for everyone” (64). Until now, mental health policy and planning have typically been informed by a “deficit approach”. This approach focuses on understanding and calculating the impact of mental ill-health on lost economic productivity and mitigation strategies primarily focus the role to be played by the health sector. Going forward, the MWI recognizes the need for an “asset approach”. This alternative approach seeks to build national prosperity through investments to promote collective cognitive and emotional wellbeing, requiring a coordinated response across health, social and economic sectors.

Beddington and colleagues, in a report of the UK Government Office for Science, proposed that the mental capital, mental health, and wellbeing of those in work “*generate the wealth that enables the wide range of public services that directly affects the mental capital and wellbeing of everyone: for example, mental health care, education, support for families, and care for our aging population*” (57). We extend this definition further and contend that a nation's Mental Wealth is generated not only from those in paid work, but more broadly from the value generated by what is termed the “social productivity” or the civil contributions of its citizens. In Australia, the value of unpaid care work (household production) not included in GDP was estimated to be A$650 billion in 2009–10, equivalent to ~51% of GDP (82). Social productivity is a broader concept that includes not only the unpaid care of the elderly, those with disability, and the education and care of children, but also the value created by volunteering, participation in community groups, environmental restoration, building community infrastructure, and other activities that are socially valued and contribute to strengthening the social fabric and cohesion of communities and nations (81). So-called “poorer” nations portrayed as lacking “wealth” may have social assets that generate significant value not currently counted, value that, if not recognized, can be undermined through traditional economic development.

Social productivity contributes to national wealth through the strong, trusting relationships it fosters between citizens, communities, business, and public institutions. The MWI is interested in how these relationships build resilience and mobilize community resources to meet both individual and collective challenges and needs. Without such mobilization, responses to crises or recovery efforts would rely to a greater extent on the state and be subject to delays due to costs and workforce constraints. The MWI is also focused on more collective notions of mental capital, mental health, and wellbeing, in recognition that the whole is greater than the sum of its parts. The Initiative acknowledges the social and economic drivers of Mental Wealth, proposing that strategic investments to enhance education, employment and economic security, social infrastructure, healthcare, physical wellbeing, early childhood development, equal access to opportunity, and other drivers, will enhance a nation's collective mental assets and have a profound impact on the resilience of communities, on a nation's economic competitiveness, and on national prosperity. In turn, we submit that as the Mental Wealth of a nation grows, so too does its resilience and capacity to nurture the drivers of Mental Wealth, perpetuating a positive feedback loop. These are propositions that will be explored through the MWI's transdisciplinary research program.

### IV. Mental Wealth: A Holistic Measure of the Value Created by Collective Human Activity

Efforts to redefine the economy and reconceptualize a prosperous society have brought about a range of measures aimed at quantifying national prosperity. These measures fall into two primary categories; those that use a monetization approach (GDP being a notable example), and those that use an index-based approach (the predominant method). Supplementary Box S1 provides a list of exemplar composite indices that attempt to capture the characteristics/elements of a prosperous society. Typically, index-based approaches rely on a process of broad consultation and development of a theoretical framework to inform the selection of indicators, normalizing (rescaling) the data for each indicator to a common unit of measure, weighting the indicators according to the extent to which they are differentially valued, and aggregating them to arrive at a composite score of national prosperity (83). There are two key issues with the index-based approach to measuring national prosperity. This approach is designed to provide a holistic view of a nation's prosperity, allow comparisons between nations and act as a narrative or “story-telling” tool (84). However, trends over time in composite index scores tend to remain relatively stable. For example, the 2021 Prosperity Index report (85) provided a summary graph of regional prosperity scores for the period 2011 to 2021 (p. 26), showing relatively stable scores for all regions and globally for the entire 10-year period. The graph of Australia's Prosperity Score over the same period also remained flat, estimated at 79.2 in 2011 and 78.8 in 2021 (86). This stability sends the wrong signal to policy makers, generating little urgency for action to improve national prosperity. The index-based approach presents a quandary; namely, that the uni-dimensionality of a composite index means that even when there is movement in the individual indicators (potentially in opposing directions), these are often masked by the composite measure, but without a composite measure, overall progress is difficult to assess. The second issue is that indices entangle within a single score the characteristics, assets, and drivers of prosperity. They give no consideration to the causal interrelationships between indicators and provide no systems analysis that could help reveal which factors should be targeted to deliver the greatest improvements in national prosperity (or indeed mitigate potential downturns in prosperity as a result of crises such as the pandemic). Rather than provide a useful decision tool for policy, they deliver an unhelpfully comprehensive list of factors contributing to national prosperity, promoting the idea that reforms, investments, and actions to increase prosperity must be so all-encompassing and long term as to render them infeasible, unimplementable, and unaffordable, reinforcing a discouraging signal to policy makers.

**Figure 1:**
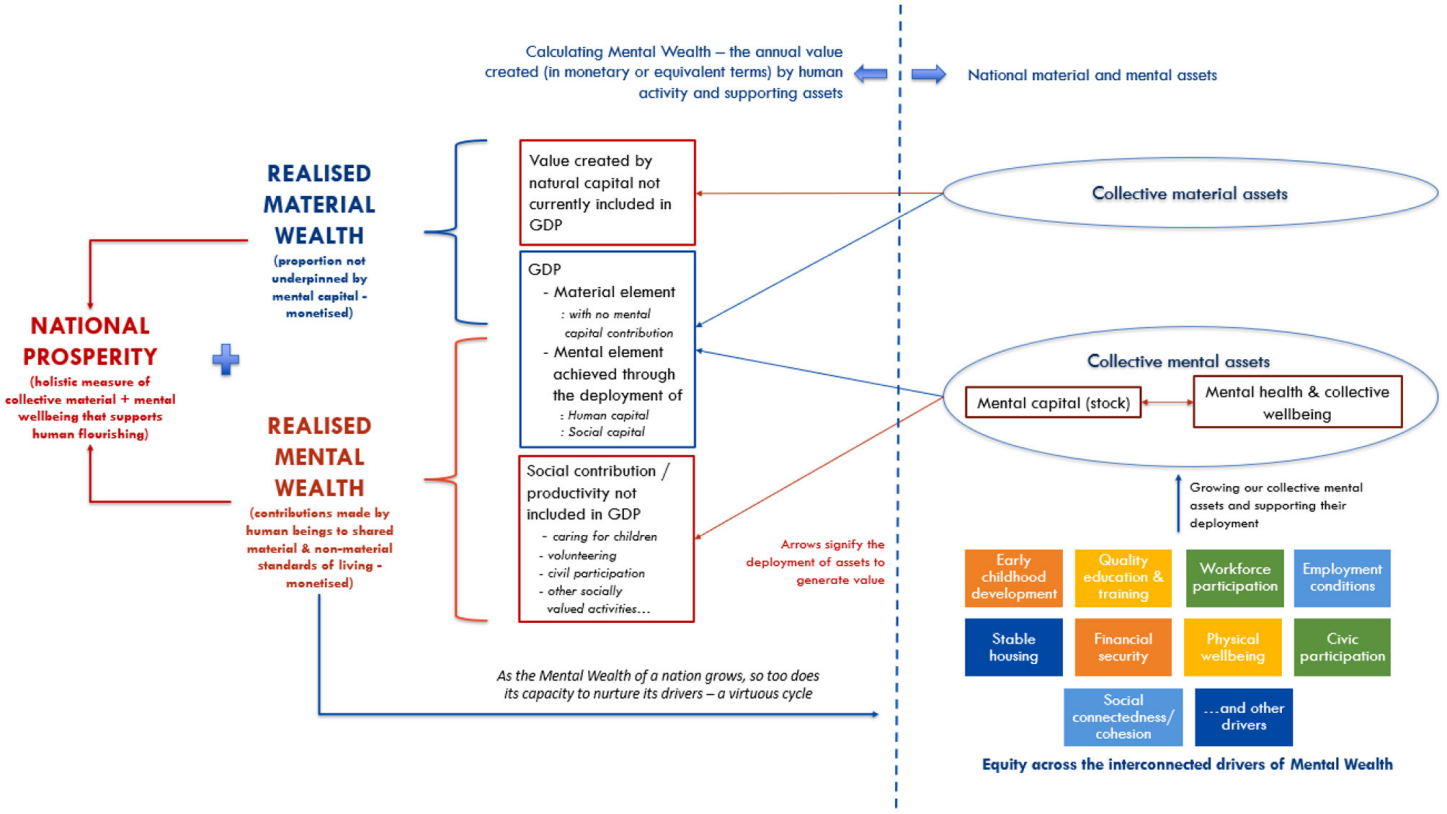
Operationalizing Mental Wealth: an initial formulation.

GDP, however, is monetized, with its quarterly and annual fluctuations reflecting expansions and contractions in a country's economy (that which is measured), prompting policy makers to take necessary action swiftly. A monetization approach to measuring Mental Wealth would have the advantage of ease of interpretability, is more likely to “wield rhetorical power” [(87), p. 902] in reframing public conceptions of prosperity, provides an impetus for policy accountability, and increases the likelihood of policy impact. In learning from and building on conventions concerning GDP, we considered two options for operationalizing Mental Wealth at a macro level. The first was a wealth accounting approach akin to the World Bank's calculation of “Total Wealth” which estimates the monetary value of produced capital (physical infrastructure), natural capital (agricultural land, forests, oil, coal, gas, mineral and other natural reserves), human capital (disaggregated by gender and type of employment), and net foreign assets (88). The Total Wealth estimate reflects the state of assets that generate GDP. Similarly, assets associated with Mental Wealth could be monetized, including mental capital, mental health, wellbeing, and social capital (infrastructure). An alternative approach would be to parallel the calculation of GDP which estimates the value created from the *deployment* of human capital and supporting assets. The latter has been selected for the emphasis it lends to a focus on societal rather than individual wellbeing further discussed below. In order to achieve its primary objective to measure, model, and forecast the Mental Wealth of nations, the MWI defines Mental Wealth as *a measure of national prosperity that captures the value generated by the deployment of collective mental assets and supporting social infrastructure* and focuses on the contributions made by human beings to material *and* non-material standards of living. Figure 1 illustrates (conceptually) the MWI's proposal to operationalize Mental Wealth. It summarizes how Mental Wealth both adds to the factors that contribute to national prosperity—but also reconfigures the traditional data items of GDP.

This approach captures and broadens GDP by applying a non-market valuation method (89) to incorporate the value of social contributions (non-monetized social productivity) as well as government and non-government expenditure on (investment in) the social infrastructure that supports productivity not already captured in GDP. In this way, Mental Wealth has the potential to become a new measure that makes explicit currently implicit sources of national prosperity. It has the potential to shift the boundary of production of the traditional economy and include the value created by activity that strengthens the fabric of society and underpins quality of life for all.

To summarize, national Mental Wealth is the monetary value of the market and non-market goods and services produced by the population over a given period, calculated as follows:


(2) $$\begin{array}{l} \text{ Mental Wealth}\;=\;\mu \text{GD}{\text{P}}_{\text{r}}\;+\;{\text{C}}_{\text{s}}\;+\;{\text{I}}_{\text{s}} \end{array}$$

where **μ** is the devaluation coefficient; the downward adjustment to *GDPr* to account for the proportion of expenditure not underpinned by mental capital (e.g., the value of mineral exports net of human input). ***GDP***_***r***_ is real GDP (for a given period) calculated using the expenditure approach. ***C***_***s***_ is the social contribution of a population (i.e., the value of the delivery of goods and services for a given period for which no contract or financial remuneration is received, including caring for children and the elderly, volunteering, environmental restoration, civil participation, unpaid professional mentoring, etc.). ***I***_***s***_ represents social capital investment, namely, the sum of government (and non-government) investment in social capital infrastructure, activities, and institutions (in a given period), not already captured in GDP. Examples include investment in surf lifesaving clubs or Rotary clubs; essentially any social infrastructure or activity that contributes to increasing community connectedness, mental capital, mental health, and wellbeing that underpin productive, creative, resilient, and thriving communities. Mental Wealth will be reported in United States Dollars, $US per capita (to allow for future between-country comparisons) and as a percent change from the previous reporting period.

Mental Wealth per capita then provides a new measure of a country's standard of living, not only in a material sense but also in a social sense, thereby reflecting the broader value generated from collective human activity. The measure of Mental Wealth values equally the contributions made to society by those not in the labor market and those who are in the labor market. It is an indicator of the health of the economy and the health of a society. Capturing both elements in a single measure will demonstrate that both are needed, since maximizing one at the expense of the other will not grow our national prosperity. Importantly, this measure also allows for the possibility of an increase in Mental Wealth even under circumstances where GDP falls, if there has been sufficient investment in things that generate true value for a thriving population. Attention to Mental Wealth keeps the pursuit of continual growth of GDP in a healthy balance with social and other valued contributions not currently captured in this metric, and in doing so, may generate strategies to grow both.

This operationalization of Mental Wealth has several additional advantages. By defining Mental Wealth as a measure of the deployment of our collective mental assets (rather than the assets themselves) the focus shifts from individual to societal wellbeing and the social environment in which people live. For example, if an individual has had all the conditions in life to support the growth of their mental capital, but they do not deploy those assets through work or unpaid contribution to society, then those assets remain with the individual. ***While mental assets may have value in their*** ***own right, it is only in their deployment to benefit society broadly (materially*** ***and/or non-materially) that they realize Mental Wealth***. This operationalization additionally draws attention not only to possible strategies to build individual mental capital, mental health and wellbeing (as primary drivers of national prosperity), but also to strategies to improve deployment of these assets for the shared benefit of society [i.e., investments to increase “civic fertility” (26), or to reduce mental capital under-utilization]. Investments in the foundational economy for example, strengthen the “infrastructure of everyday life” (31), enabling the deployment of collective mental assets, because these fundamental goods and services underpin any thriving society. Especially initially, the Mental Wealth measure will be imperfect, but refinements can be made over time and most importantly, the measure will make visible important human productive activity not captured in GDP. The measure will also provide an objective baseline against which to assess changes over time, forecast future trajectories, simulate the likely impacts of strategic reforms and investments, and encourage policy makers to take proactive actions to grow national prosperity.

### V. The Importance of a Systems Lens

The concept of Mental Wealth and its drivers builds on decades of research into the social determinants of mental health (90), on how to measure and build mental and social capital (63, 91–94), and on progressive economic theory (29, 31, 33, 46, 55, 95–98). Beyond this, the Mental Wealth Initiative (MWI) has been grappling with how to connect these literatures, how to quantify and better understand the dynamics that drive Mental Wealth and its contribution to a thriving society, and how to forecast the likely trajectory of a nation's Mental Wealth. In doing so, the MWI aims to make a more compelling case for governments to invest strategically in policies and programs that build mental capital and foster the mental health and wellbeing of the populations and communities they serve.

The factors that drive Mental Wealth are not independent, they are interdependent, with threshold effects and feedback loops that can result in virtuous cycles and vicious cycles (95). For example, adverse economic conditions and industrial relations reforms aimed at enhancing business flexibility and creating jobs can see labor markets increasingly characterized by insecure, fixed-term, temporary and casualized work (99–101). These sorts of precarious employment arrangements can undermine the economic security of households, increase social dislocation and isolation (102, 103), increase parental stress and marital tension (104), and increase rates of domestic violence (105, 106), and child abuse and neglect (107, 108). Adverse experiences early in life can have profound effects on mental health and development (109, 110), leading to increasing rates of psychological distress, substance misuse, physical health issues, behavioral challenges, and suicidal behavior (111–116). All this can result in significant functional impairment further eroding mental and social capital, labor market performance (117–119) and contributions to social productivity.

These characteristics of interconnectedness and feedback loops challenge traditional analytic methods. Complex systems science provides the transdisciplinary framework and analytic methods to study the interactions between health, economic, and social systems. It enables simulation to forecast the impact of policy and investment decisions, and as highlighted in a recent *Nature* commentary (120), systems modeling (Box 2) can inform responses to the social and economic impacts of COVID-19 on mental health. Systems modeling provides an important tool in the repertoire that helps decision makers explore the systemic consequences of policy levers in shaping more inclusive and sustainable social and economic development as we reconstruct the Mental Wealth of nations in the post-pandemic era.

Box 2 **What is systems modeling?**

Systems modeling applies mathematical, statistical, and computational modeling techniques to represent the structure and behavior of complex human, health, and social systems (121). Systems models are simplifications of real-world systems capable of reproducing many observed data patterns and forecasting future trajectories. They are ideal for allowing insights and data from different disciplines and domains of reality to be brought together. Their ultimate purpose is to examine the potential consequences (including unintended consequences) of proposed interventions before they are implemented in the real world. System dynamics (SD) models (also known as “stock and flow” models) are a subset of systems models that aim to capture, at an aggregate level, stock accumulations driven by flow dynamics (122). SD models have long been used in many branches of the physical and life sciences (123). In the business, engineering and finance sectors, systems modeling has been deployed to support strategic planning, reduce supply chain instability, improve operational and allocative efficiency, improve public safety, and determine which products and services will deliver the best returns on investment, in which markets, at what time, and at what price (124, 125). Systems modeling was also recently used to inform public health responses to COVID-19 in several countries.

The University of Sydney's Brain and Mind Centre (BMC) has an established program of systems modeling and simulation to support decision making for mental health service planning, suicide prevention, and mitigating the social and economic impacts of COVID-19 on mental health (120, 126). To catalyze practical change in local areas, we must bring people along on the journey of learning with us, hence BMC invests the time and effort required to ensure model building is an open, transparent, participatory process. This process brings together the best available research evidence and data, as well as expert, local and experiential knowledge (127) (https://www.youtube.com/watch?v=G9DwKEBLBC4).

**Figure 2:**
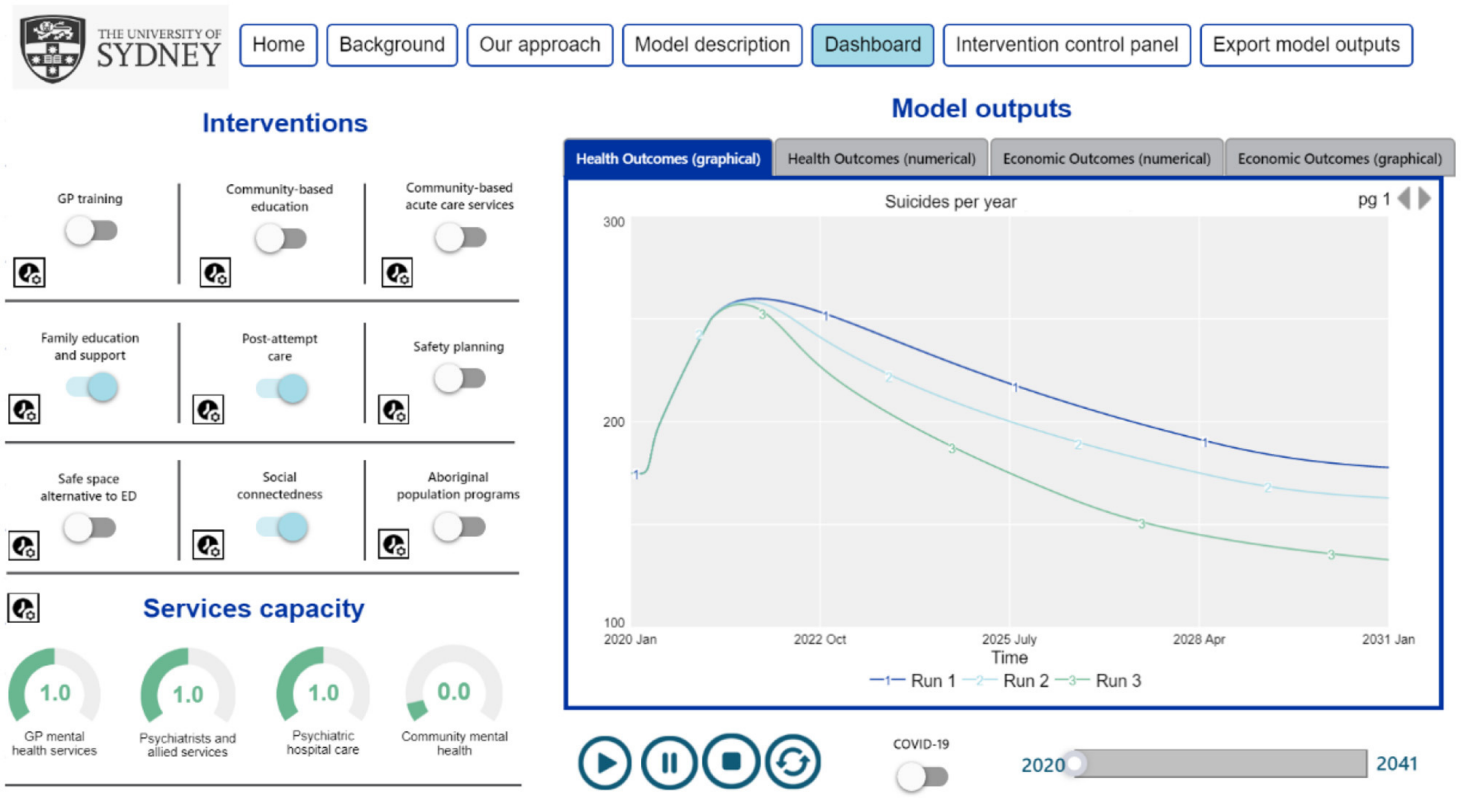
An example interactive interface demonstrating how stakeholders can engage with systems models as decision support tools; simulating the likely impacts of policies and initiatives before investing in them.

What emerges from this process is a computer simulation model that not only forecasts future trajectories of mental health and economic outcomes but allows decision makers and stakeholders to ask “what if” questions. Interactive model interfaces allow them to turn policies and initiatives on and off, scale services up and down, stagger their implementation and test the impacts of different combinations of interventions against a baseline of business as usual (Figure 2). These transparent, interactive models can then be used to facilitate sophisticated, informed discussion among decision makers and stakeholders about what is needed to achieve shared outcomes over what timeframe, and with what compromises (120, 127, 128).

### VI. Dynamic Modeling of Mental Wealth

**Figure 3:**
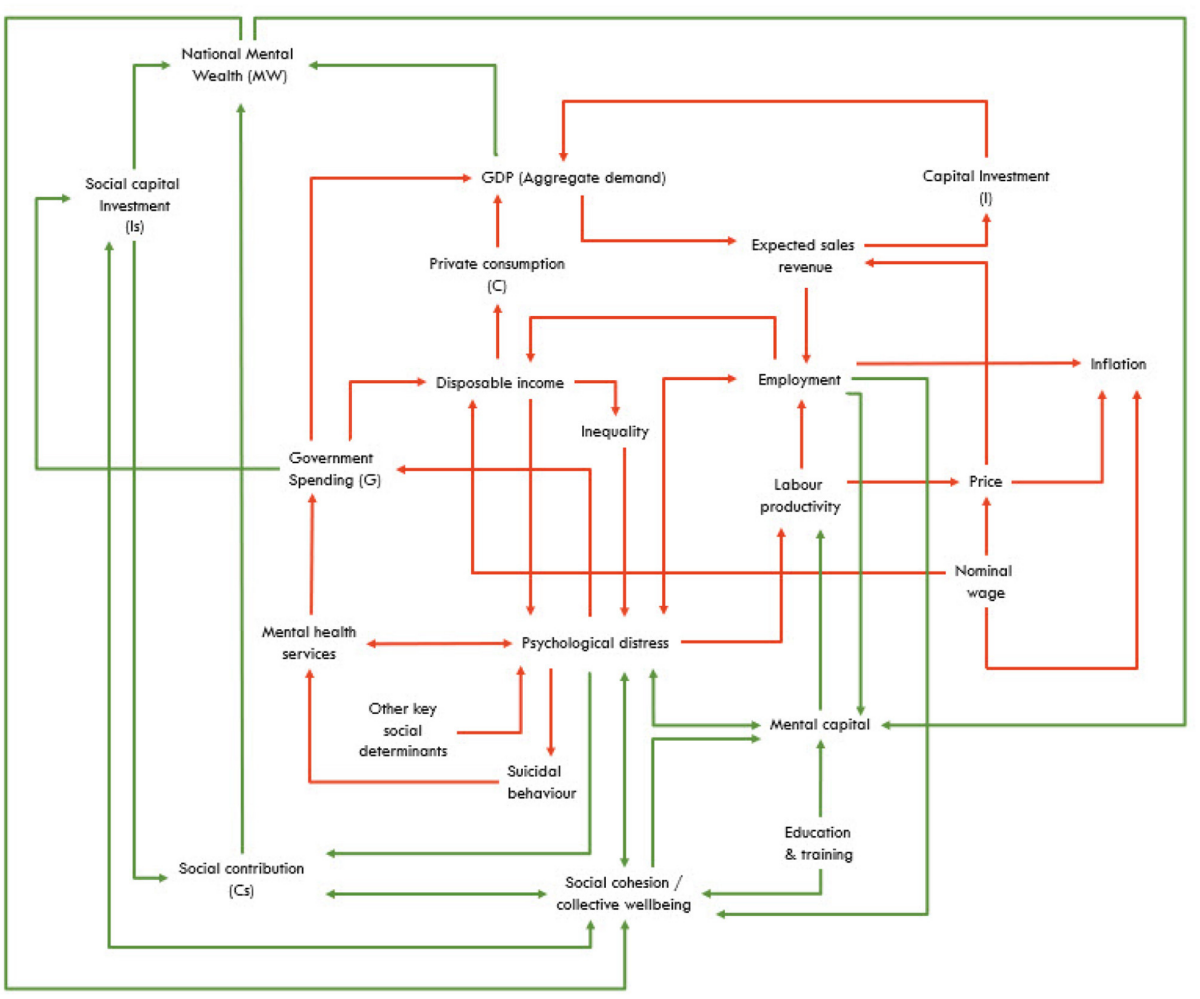
A high-level overview of the hypothesized (macro) causal structure and pathways of the proposed dynamic Mental Wealth model. Arrow colors reflect Phase 1 (red) and Phase 2 (green) of model development.

The MWI is exploring the feasibility of estimating and forecasting national Mental Wealth and understanding the extent to which government policy-mediated changes in the economic and social environment can enhance national prosperity. This empirical work will draw on our extensive experience in systems modeling (127, 129–138), and will build on work already undertaken (139, 140) and currently underway (126) to model the interacting social, economic, and health system drivers of mental health. In brief, the next steps to developing an initial Australian prototype Mental Wealth system dynamics model are as follows:

**Phase 1: To endogenise the macroeconomy** into our existing (refined) national system dynamics model of mental health. Figure 3 provides a preliminary high-level overview of the causal structure and pathways of the proposed model, with arrows denoting unidirectional or bidirectional relationships between components that will provide a starting point for investigation. The model will also include a population component, capturing changes over time in the size and composition of the population resulting from births, migration, aging, and mortality (not shown). The direct constructs of the GDP equation (expenditure approach) are captured, as are the interrelationships between the broader drivers of GDP (red arrows) based on current understanding of the interactions within the economy.

**Phase 2: To endogenise the broader essential constructs and dynamics that drive Mental Wealth** including mental capital, social capital infrastructure investment, and social contribution (productivity). Figure 3 highlights how these vital constructs and their interrelationships with social cohesion, mental capital, mental health, inequality, and other factors will be captured in the causal hypothesis. While not shown in the Figure, each construct/component of the model will be elaborated through detailed stock and flow structures. The model will capture changes over time (dynamics) within each component and between the components of the model, including feedback loops. Systems modeling is an iterative process of hypothesis building and testing; therefore, the causal interrelationships shown in Figure 3 are likely to evolve and are provided herein for illustrative purposes only.

System dynamics models draw on a broad range of evidence and data sources to posit, quantify, test, and validate a causal structure and parameters that underly observed data patterns (141). Where possible, model parameter values will be derived directly from the research program of the MWI and its collaborating institutions, published research from a broad range of disciplines, publicly available aggregate data sets (e.g., from the Australian Bureau of Statistics, Australian Institute of Health and Welfare, state and territory departments of education, health and social services, the Reserve Bank of Australia), and government reports. Missing parameter values will be estimated via constrained optimization (model calibration). Model construction and analysis will be performed using Stella Architect (www.iseesystems.com). The model will be validated by (i) testing whether the outputs of the model can replicate historic data across a range of key indicators including time series of GDP per capita, labor productivity, government expenditure, the proportion of the population not in employment, education, or training (NEET), and key mental health outcomes such as psychological distress, psychiatric hospitalizations, and suicidal behavior; and (ii) ensuring face validity of the model structure and performance among stakeholders working in or interacting with different parts of the system. Additional primary data collection may be required over time to strengthen the model parameter estimates, test assumptions, and hence further verify the causal hypothesis. Both phases will be undertaken using a transparent stakeholder consultation process that will draw on expertise in mathematics, biostatistics, epidemiology, psychology, psychiatry, social science, policy, economics, business, and lived experience.

#### Policy Testing and Sensitivity Analyses

A range of policies, initiatives, and system reforms will then be integrated into the model. This will facilitate scenario testing and allow *a priori* exploration of the impacts of efforts to strengthen the education sector [e.g., exploring return on investment in early childhood education vs. post-secondary job training (142)], the mental health system (e.g., workforce expansion and training, IT-facilitated service delivery), social infrastructure (e.g., the universal basic income, four day work week, investment in greenspace, libraries, community markets), as well as test macroeconomic reforms (e.g., job guarantee, taxation reform, fiscal stimulus, working time reduction supported by work-sharing). Systems models not only facilitate a better understanding of the behavior of a system as it currently is but can also simulate reforms (changes to structure and pathways) to explore the system as it would need to be to foster the Mental Wealth of current and future generations.

Simulation will allow comparison against a baseline (business-as-usual) of the impact of a range of cross-sector strategies on GDP and national Mental Wealth and provide decision support capability for future investments and actions to foster Mental Wealth. Sensitivity analyses will be performed to assess the impact of uncertainty in estimates of the direct effects of each intervention (policy, initiative, or reform), and other uncertain parameters on the simulation results. All simulations will be projected over a 10–20-year period to allow impacts of major reforms to be fully realized, as well as to encourage a long-term strategic outlook in assessing the value of investment decisions. The MWI will also work with key agencies to strengthen data ecosystems to facilitate continuous feedback between real-world and modeled systems. This will enable refinement (and extension) of the Mental Wealth model over time and deliver greater confidence in forward projections of the impact of policy responses. Once established, this prototype could form the basis of subnational applications to better understand the distribution of Mental Wealth across regions and the potential differential impacts of policies, reforms, and initiatives to foster it.

## Discussion

### From Research to Action

While further research and public education concerning Mental Wealth are important, increasing new knowledge and learning in themselves are not enough to break with current trajectories of development and reconstruct more resilient economic, health and social systems. Many of the factors undermining Mental Wealth are complex and are the product of decades of social neglect or deprivation. The economic recovery of nations in the post-pandemic era, based on (re)building Mental Wealth, will require nothing less than the combined instruments of science, policy, politics, public resolve, social legislation, and international cooperation to shift us onto a new path. The vision of the MWI is to provide the knowledge, the tools, and the forums needed to enact change.

The ideas informing the MWI are shared by a wide and diverse range of researchers and policy makers. Important work over the last two decades has been undertaken by the human wellbeing and social determinants of health movements. The occupational health and safety realm is increasingly concerned with work as a site of wellbeing, and not just as a place where harm needs to be minimized. In the humanities and social sciences, the capabilities approach has spawned a diverse and extensive literature. While there have been great advances in the realms of research and policy proposals, implementing effective change in everyday life and influencing government funding priorities has been relatively unimpressive. Currently efforts are fragmented. If we want to turn vicious cycles into virtuous cycles, we need to create new institutional capacity and come together across academia, industry, and community settings to help rebuild the cultural, political, and social character of nations. Therefore, the MWI is committed to providing a mechanism to facilitate the exchange of knowledge and ideas, to harness collective efforts, networks, and resources, and to coordinate advocacy and action both nationally and internationally.

Like other countries, Australia has shown in its response to the COVID-19 pandemic that rapid, dramatic changes are possible to protect collective wellbeing. As we transition to the post-pandemic reconstruction phase, it is vital we use this global crisis as a turning point, a moment to rethink what we, as a society really value. The MWI is informed by the simple idea that optimal economic and social development requires the creation of environments where all individuals can reach their full potential and contribute productively to society. Creating such environments requires broad, non-linear systems thinking, advanced analyses, cross-disciplinary cooperation, and the harnessing of collective knowledge, networks, resources, and fellowship. It is this combination that will set us on the path to achieving cohesive, well-functioning societies, capable of facing future global challenges with collective resilience and unity.

## Author Contributions

JO, JB, AS, and IH: manuscript concept and drafting. All authors: critical revision of manuscript for important intellectual content. All authors contributed to the article and approved the submitted version.

## Funding

This work was primarily supported by the Mental Wealth Initiative funded by the University of Sydney, and additionally supported by philanthropic funding to the Brain and Mind Centre, University of Sydney. PM was supported by the UK Prevention Research Partnership (SIPHER Consortium, MR/S037578/1 and MR/S037578/2) and the Systems Science in Public Health Programme (MRC: MC_UU_00022/5 and CSO: SPHSU20). This work is additionally supported (in kind) by the World Economic Forum's Global Future Council for Mental Health, whose leadership, networks, and guidance are providing a valuable springboard for global applications of systems modeling and simulation to inform effective strategies for improving population mental health and mental wealth.

## Conflict of Interest

JO is Co-Director of the Mental Wealth Initiative and Head of Systems Modelling, Simulation and Data Science at the University of Sydney's Brain and Mind Centre. She holds a non-salaried position as Managing Director of Computer Simulation and Advanced Research Technologies (CSART), a registered charity. She also serves on the World Economic Forum's Global Future Council on Mental Health. JB is Professor in the Discipline of Business Information Systems in the University of Sydney Business School. He is Co-Director of the Mental Wealth Initiative. He is also on the advisory board (voluntary) for two non-government organizations: (i) the People Development Council of Dairy Australia (the peak organization for dairy farmers and processes in Australia), and (ii) the Centre for Future Work. AF is Chair of Mind Australia (an NGO) and a Board Member of the Haven Foundation (an NGO). PD is Co-Chair of the World Economic Forum's Global Future Council on Mental Health and has received travel support from the World Economic Forum. He serves on the boards of Live Love Laugh Foundation and Goldie Hawn Foundation which work to enhance mental health. He has received the following: research grants from the NIH, DOD, Eli Lilly, Avanir, Avid, Cure Alzheimer's Fund, Karen L Wrenn Trust and Steve Aoki Foundation; advisory fees from Vitakey, Lumos Labs, Otsuka, Apollo, Transposon, Sermo, and Neuroglee; stock in Evidation Health and UMethod (whose products are not discussed here). His also co-inventor, through Duke, of patents for diagnosis or treatment of various disorders. AP holds a non-salaried position as an Independent Director of Computer Simulation and Advanced Research Technologies Ltd. (CSART), a registered charity. IH serves on the World Economic Forum's Global Future Council on Mental Health. He was an inaugural Commissioner on Australia's National Mental Health Commission (2012–18). He is Co-Director, Health and Policy at the Brain and Mind Centre (BMC), University of Sydney, Australia. The BMC operates an early-intervention youth services at Camperdown under contract to *headspace*. He is also the Chief Scientific Advisor to, and a 5% equity shareholder in, InnoWell Pty Ltd. InnoWell was formed by the University of Sydney (45% equity) and PwC (Australia; 45% equity) to deliver the $30M Australian Government-funded Project Synergy (2017–20; a 3-year program for the transformation of mental health services) and to lead transformation of mental health services internationally through the use of innovative technologies. The remaining authors declare that the research was conducted in the absence of any commercial or financial relationships that could be construed as a potential conflict of interest.

## Publisher's Note

All claims expressed in this article are solely those of the authors and do not necessarily represent those of their affiliated organizations, or those of the publisher, the editors and the reviewers. Any product that may be evaluated in this article, or claim that may be made by its manufacturer, is not guaranteed or endorsed by the publisher.
