# Supplementary material for: Measuring, Modeling, and Forecasting the Mental Wealth of Nations
Source: Front Public Health. 2022 Jul 28;10:879183. doi: 10.3389/fpubh.2022.879183 (PMC9368578; doi:10.3389/fpubh.2022.879183)
Supplement: Supplementary file 1 [file Table_1.PDF]

Table S1: Estimating national prosperity: Recent proposals for overcoming GDP's limitations

**Nature of proposed change to GDP**	**Leading research programs + recent/key publications**	**Key categories/data items of interest**		**Strengths**	**Weaknesses**
**Adding data items**	'Beyond GDP'<br>- (Stiglitz, Sen, and Fitoussi 2010)<br>- (OECD 2011)<br>- (Durand 2015)<br>- (Stiglitz, Fitoussi, and Durand 2018b, a)<br>- (OECD 2020)<br><br>Human Development Index<br>- (Haq 2009)<br>- (United Nations Development Program 2020)<br>- (Conceicao, Kovacevic, and Mukhopadhyay 2021)	OECD wellbeing framework<br>- current wellbeing<br>: quality of life<br>- Health status, work-life balance, education+ skills, social connectedness, civic engagement/governance, environmental quality, personal security, subjective well being<br>: material conditions<br>- income + wealth, jobs + earnings, housing<br>- Resources for future wellbeing<br>: Physical, economic, human + social capital	Human Development Index<br>- Foundations in the capabilities approach<br>- Key data items concern<br>: long + healthy life,<br>: Knowledge/Education<br>: material living standards (GNI per capita PPP)<br><br>- Extensive work on inequality (income and gender based)<br>: multi-dimensions (health, schooling, access to water, electricity, sanitation)	Increases immensely the availability of quality data, especially time series on a host of key variables that vital to the quality of life<br><br>HDI is particularly comprehensive in terms of global coverage and capturing hard data items on health, education, and material life.<br><br>- Reconfigurations for inequality multi-dimensional	GDP as currently defined untouched, leaving definitional flaws in constitutive data items unaddressed.<br><br>Alternative indices lack monetisation or equivalent for valuation.<br><br>Aggregation of ordinal indexes results in slow moving aggregations of limited use.<br><br>Formulation of data items as separate => connections + feedback between them is neglected (De Smedt, Giovannini, and Radermacher 2018)
**Re-configuring data items**	World Inequality Database<br>- (Alvaredo et al. 2018)<br><br>Foundational Economy<br>- (Froud et al. 2018)	Distributions of income + wealth<br>- functional distribution/factor shares of national income<br>- personal distributions (quartiles, deciles, percentiles)<br>- household expenditures	Foundational liveability index<br>- E.g., residual household income after expense on material foundations of life (housing, transport, utilities	The work on distribution has profoundly changed the way 'growth' is now viewed.<br><br>Liveability index pierces complacency around growth maximisation as unambiguously 'better'	While strong on income + financial wealth dimension of prosperity, leaves core remaining core data items of GDP unchallenged.
**Refining/ Recasting core data items**	Successful Societies<br>- (Hall and Lamont 2009)<br><br>Mental Wealth<br>- (The Government Office for Science 2008)<br>- (Beddington et al. 2008)	Successful societies categories<br>- Material conditions – social conditions defining distinction<br>- Challenges – capacities nexus	Mental wealth categories<br>- Material wealth – mental wealth is the threshold defining distinction<br>- Mental capital – Mental wellbeing/connectedness nexus is central to generating mental wealth.	Key ideas highlight the inadequacies of how core GDP categories defined especially<br>- mental/social are often integral to 'priced' entities but not recognised. In the non-market sector both are totally overlooked<br>- need to understand relational/connected dimension of items of interest	While provide great ideas, little has been done to operationalise them in a way that is of practical use in both supplementing as well as overcoming problems within core categories of GDP.

Box S1: Exemplar indices of a prosperous society

- **The WEF's Inclusive Development Index** (World Economic Forum 2018) – measures how countries perform on 11 dimensions of economic progress in addition to GDP. It has 3 pillars; growth and development; inclusion; and intergenerational equity
- **OECD Better Life Index** (Organisation for Economic Cooperation and Development 2020) – captures dimensions of material living conditions and quality of life including housing, income, jobs, community, education, civic engagement, health, life satisfaction, safety, and work-life balance.
- **Social Progress Index** (Social Progress Imperative 2018) – ranks 168 countries on social progress by combining 53 social and environmental outcome indicators to calculate an overall score for these countries, based on tiered levels of scoring that include measures in health, safety, education, technology, rights, etc.
- **Global Green Economy Index** (Dual Citizen LLC 2021) – measures the green economy performance of 130 countries (using quantitative and qualitative indicators) to assess how well each country performs on four key dimensions: leadership & climate change, efficiency sectors, markets & investment and the environment.
- **The Doughnut economics framework** (Raworth 2017) – capturing the social foundation including food, water, health, energy, education, housing, income & work, equity, networks; as well as ecological indicators including climate change, ocean acidification, pollution, biodiversity loss, land conversion and ozone depletion.
- **The UN Human Development Index** (United Nations Development Programme 2020) – a composite index of performance on three dimensions of human development; life expectancy, access to knowledge / education, and standard of living. Developed over 30 years ago, the purpose of the index was to promote person-centered policies. The HDI has since been adjusted to account for inequality.
- **The Legatum Prosperity Index** (Legatum Institute 2021) – a framework that assesses countries on economic and social wellbeing and their promotion of human flourishing. The Index consists of 12 'Pillars of Prosperity,' namely, safety and security, personal freedom, governance, social capital, investment environment, enterprise conditions, infrastructure and market access, economic quality, living conditions, health, education, and natural environment.

References

Alvaredo, F., L. Chancel, T. Picketty, S. Emmanuel, and G. Zucman. 2018. "Distributional national accounts." In *For Good Measure. Advancing Research on Well-being Metrics beyond GDP*, edited by J. Stiglitz, Fitoussi J-P. and M. Durand, 143-162. Paris: OECD.

Beddington, J., C. L. Cooper, J. Field, U. Goswami, F. A. Huppert, R. Jenkins, H. S. Jones, T. B. Kirkwood, B. J. Sahakian, and S. M. Thomas. 2008. "The mental wealth of nations." *Nature* 455 (7216):1057-60. doi: 10.1038/4551057a.

Conceicao, P.M., M. Kovacevic, and T. Mukhopadhyay. 2021. "Human Development: A Perspective on Metrics." In *Measuring Human Capital*, edited by B. Fraumeni, 83 - 117. San Diego: Academic Press.

De Smedt, M., E. Giovannini, and W. Radermacher. 2018. "Measuing sustainability." In *For Good Measure. Advancing Research on Well-being Metrics beyond GDP*, edited by J. Stiglitz, Fitoussi J-P. and M. Durand, 241 - 282. Paris: OECD.

Dual Citizen LLC. 2021. "The Global Green Economy Index." Dual Citizen LLC, accessed 08/12/2021.

Durand, Martine. 2015. "The OECD Better Life Initiative: How's Life? and the Measurement of Well-Being." *Review of Income and Wealth* 61 (1):4-17. doi: https://doi.org/10.1111/roiw.12156.

Froud, J., C. Haslam, S. Johal, N. Tsitsianis, and K. Williams. 2018. Foundational Liveability: rethinking territorial inequalities. In *Foundational Economy Collective: Working Paper No.5*. https://foundationaleconomycom.files.wordpress.com/2018/12/foundational-livability-wp-no-5-fe-collective.pdf.

Hall, P., and M. Lamont. 2009. *Successful Societies: How Institutions and Culture Affect Health*. New York, NY: Cambridge University Press.

Haq, U. 2009. "The birth of human development." In *Handbook of Human Developent: concepts, measures, and policies*, edited by S. Fuk-Par and S. A. K. Kumar, 127-137. New Delhi: OUP.

Legatum Institute. 2021. The Legatum Prosperity Index 2021: A tool for transformation. London: https://www.prosperity.com/download_file/view_inline/4429: Legatum Institute.

OECD. 2011. How's Life? Measuring well being. Paris: OECD.

OECD. 2020. How's life? 2020 Measuring well-being. Paris: OECD.

Organisation for Economic Cooperation and Development. 2020. "How's Life? 2020." OECD Publishing, accessed 08/12/2021.

Raworth, K. 2017. "A Doughnut for the Anthropocene: humanity's compass in the 21st century." *Lancet Planet Health* 1 (2):e48-e49. doi: 10.1016/S2542-5196(17)30028-1.

Social Progress Imperative. 2018. "Social Progress Index: From data to impact." Social Progress Imperative, accessed 08/12/2021.

Stiglitz, J., J. Fitoussi, and M. Durand. 2018a. *Beyond GDP: Measuring What Counts for Economic and Social Performance*. Paris: OECD Publishing.

Stiglitz, J., J. Fitoussi, and M. Durand. 2018b. *For Good Measure: Advancing Research on Well-being Metrics Beyond GDP*. Paris: OECD Publishing.

Stiglitz, J., A. Sen, and J-P. Fitoussi. 2010. *Mismeasuring Our Lives: Why GDP Doesn't Add Up. Report of the Commission on Measurement of Economic Performance and Social Progress*. New York: New press.

The Government Office for Science. 2008. Foresight Mental Capital and Wellbeing Project (2008). Final Project report. London: https://assets.publishing.service.gov.uk/government/uploads/system/uploads/attachment_data/file/292453/mental-capital-wellbeing-summary.pdf: The Government Office for Science.

United Nations Development Program. 2020. Human Development Repoprt 2020 - The next frontier: Human development and the Anthropocene. New York: UNDP: http://hdr.undp.org/sites/default/files/hdr2020.pdf.

United Nations Development Programme. 2020. "Human Development Index (HDI)." UNDP, accessed 07/02/2022. https://hdr.undp.org/en/content/human-development-index-hdi.

World Economic Forum. 2018. The Inclusive Development Index 2018. Geneva, Switzerland: World Economic Forum.
